# Prevalence of Perinatal Depression and Anxiety in Both Parents: A Systematic Review and Meta-analysis

**DOI:** 10.1001/jamanetworkopen.2022.18969

**Published:** 2022-06-24

**Authors:** Kara L. Smythe, Irene Petersen, Patricia Schartau

**Affiliations:** 1 Department of Primary Care and Population Health, Institute of Epidemiology and Health Care, University College London, London, United Kingdom

## Abstract

**Importance:**

New and expectant parents experience perinatal mood disorders, with consequences to parenting ability, bonding with the neonate, interpersonal relationships, and health and well-being of parents. Research shows that maternal and paternal perinatal mood disorders are associated, but no recent systematic review has addressed the prevalence of perinatal mood disorders in both mothers and fathers (parental dyad).

**Objective:**

To examine the prevalence of perinatal mood disorders in parental dyads and identify factors associated with perinatal mood disorders in parental dyads.

**Data Sources:**

Ovid (MEDLINE, Embase, and PsycINFO) and Web of Science were searched from January 1, 1990, to June 8, 2021, for observational studies reporting on the prevalence of perinatal depression or anxiety in a parental dyad.

**Study Selection:**

Studies reporting the prevalence of anxiety or depression in both members of a parental dyad were included, with diagnosis according to established criteria (*Diagnostic and Statistical Manual of Mental Disorders* [Fifth Edition], *International Classification of Diseases, 11th Revision*) or use of validated screening tools.

**Data Extraction and Synthesis:**

Prevalence data were extracted in accordance with Preferred Reporting Items for Systematic Reviews and Meta-Analyses guidelines. Data were analyzed in subgroups: antenatal depression, early postnatal depression (0-12 weeks), late postnatal depression (3-12 months), and perinatal anxiety. Pooled prevalence was calculated using a random-effects meta-analysis model. Quality assessment was performed using Joanna Briggs Institute Appraisal Checklist for Studies Reporting Prevalence Data. Data were analyzed in June 2021.

**Main Outcomes and Measures:**

Prevalence of perinatal anxiety and perinatal depression in parental dyads.

**Results:**

Twenty-three studies were included, with data from 29 286 couples. The pooled prevalence of antenatal depression in both parents was 1.72% (95% CI, 0.96%-2.48%; *P* < .001). The prevalence of early postnatal depression (up to 12 weeks post partum) was 2.37% (95% CI, 1.66%-3.08%; *P* < .001) and the prevalence of late postnatal depression (3-12 months post partum) was 3.18% (95% CI, 2.3-4.05; *P* < .001). Only 3 studies reported on perinatal anxiety in both parents, precluding a quantitative analysis.

**Conclusions and Relevance:**

In up to 3.18% of couples, both parents may concurrently experience perinatal depression. Perinatal health care must consider the mental health needs of parents, both as individuals and as a parental dyad. Further research is needed to examine outcomes in families where both parents experience perinatal mood disorders.

## Key Points

**Question:**

How often do both mothers and fathers (parental dyad) experience perinatal mood disorders?

**Findings:**

In this systematic review and meta-analysis of 23 studies with 29 286 couples, up to 3.18% of parental dyads (both mothers and fathers) experienced perinatal depression, and prevalence was higher in the late postnatal period (3-12 months). There were insufficient data on parental perinatal anxiety to draw any conclusions.

**Meaning:**

These findings suggest health care workers caring for new or expectant parents should be aware that both parents can concurrently experience perinatal mood disorders, with consequences for their health and well-being as well as that of their infant.

## Teaser

This systematic review and meta-analysis evaluates the prevalence of perinatal depression in both mothers and fathers.

## Introduction

Common mental disorders such as anxiety and depression are associated with morbidity for new and expectant parents. Meta-analyses of maternal depression in high-income countries estimate a prevalence of 11% during pregnancy, and 13% in the postnatal period.^1^ A meta-analysis of paternal depression with data from 21 countries estimated a prevalence of 9.76% during pregnancy and 8.75% during the first postnatal year.^2^ Prevalence of maternal anxiety varies depending on which disorders are included in the estimate but can be as high as 13%.^3^ Paternal perinatal anxiety is associated with maternal depression, and the odds of paternal anxiety increase by more than 3-fold when mothers are depressed.^4^

Psychosocial factors associated with risk for maternal perinatal mood disorders include early life stressors, limited social support, and exposure to intimate partner violence. Pregnancy-related factors, such as unintended pregnancy or somatic symptoms, such as low back pain or nausea and vomiting in pregnancy, also increase the risk of perinatal depression.^1,5,6^ Factors such as lower levels of education, unemployment, low social support, or marital distress increase the risk of paternal perinatal mood disorders.^4,7^ A history of mood disorders also increases the risk of perinatal depression or anxiety for both mothers and fathers.^1,7^ However, it is not currently known whether there are factors that increase the risk of perinatal mood disorders occurring concurrently in both members of the parental dyad (mothers and fathers).

Maternal and paternal mental health are associated,^8^ and common mental disorders may be experienced concurrently by both members of the parental dyad. Mood disorders in 1 parent may impact the other parent, and there is evidence that paternal depression leads to increased symptoms of depression in mothers during pregnancy and in the first 6 postpartum months.^9^ Parental perinatal mood disorders are associated with adverse pregnancy outcomes, impaired bonding with the newborn, and behavioral problems in their children.^1,10,11^ Co-occurrence of mood disorders in both parents may amplify these negative outcomes; however, prevalence data are lacking. This information is necessary to inform health care priority-setting and facilitate a move toward a family-centered model of care that better serves mothers and fathers as they transition to parenthood. The aims of this systematic review were to (1) examine the prevalence of perinatal mood disorders in both parents, and (2) identify factors associated with increased risk of mood disorders for both members of the parental dyad (mother and father).

## Methods

The protocol was written according to Preferred Reporting Items for Systematic Reviews and Meta-analyses (PRISMA) reporting guideline.^12^ The study was registered in advance on PROSPERO (CRD42021252140). This systematic review was exempt from institutional review board (IRB) (ethical approval at University College London) as it involves data which are publicly available and the included articles received IRB/ethical approval.

### Literature Search

A systematic search was performed on Ovid (MEDLINE, Embase, and PsycINFO) and Web of Science and completed on June 8, 2021. The search strategy combined outcome(s) of interest (depression or anxiety), population of interest (mothers and fathers), and time (perinatal or antenatal or postnatal or postpartum or antepartum or pregnancy).

### Study Selection

Observational studies published in English between January 1, 1990, and June 8, 2021, were eligible for inclusion. This date range captures studies published after the Edinburgh Postnatal Depression Scale (EPDS) came into regular use. Intervention studies were not included as the narrower inclusion criteria might limit external validity.

Titles were screened, and irrelevant articles were excluded. A second reviewer screened 50% of the abstracts with 99% consensus. Disagreements were resolved by discussion. Abstracts that met the criteria were selected for full-text review. The screening, full-text review and data extraction were performed using Covidence Web-based software version 2852 (Covidence).

#### Participants

Studies reporting data for parental dyads (mothers and fathers) were eligible for inclusion. Studies focusing exclusively on special populations (eg, adolescent pregnancy, premature delivery, and parents experiencing a stillbirth) were excluded. These pregnancy complications likely represent differential risk for perinatal mood disorders, and inclusion of these populations would limit generalizability. Case-control studies with a clearly defined low-risk control group were eligible, provided necessary outcome data were available.

#### Outcome Measures

Studies reporting the incidence or prevalence of perinatal mood disorders, or providing sufficient data to permit calculation, were included. Diagnoses had to be based on clinical criteria (*Diagnostic and Statistical Manual of Mental Disorders* [Fifth Edition] or *International Classification of Diseases, 11th Revision*) or use of a validated screening questionnaire to identify those at risk (eg, EPDS). Diagnoses must have been rendered during pregnancy or in the first 12 months after childbirth, and mothers and fathers had to be assessed as a parental unit. Review articles or studies performed solely for validation of a screening tool were not included. Full inclusion and exclusion criteria can be found in eAppendix 1 in the Supplement.

### Quality Assessment

Study quality was assessed using Joanna Briggs Institute Critical Appraisal for Studies Reporting Prevalence Data.^13,14^ This tool covers essential domains of population, measurement, and statistical approach.^15^ If sample size calculations were not reported, then the sample size was assessed according to whether the study was powered to detect a 15% prevalence of maternal depression within a 5% margin of error and 95% confidence. This value was chosen as it is the upper limit of prevalence reported in the literature.^1^ Thus, a minimum sample size of 196 couples met this criterion.

### Statistical Analysis

Prevalence values of depression or anxiety in mothers, fathers, and couples were extracted. The 95% CIs were calculated for all prevalence values and these data were imported into StataMP statistical software version 16 for Mac (StataCorp) for meta-analysis. Data were analyzed in June 2021.

Outcome data were arranged in 4 subgroups: antenatal depression, early postnatal depression (up to 12 weeks [3 months] after delivery), late postnatal depression (>3-12 months after delivery), and anxiety. Postnatal depression in the year following childbirth was subdivided to reflect what is commonly considered the fourth trimester (up to 12 weeks after delivery).^16^ The pooled prevalence of antenatal, early postnatal, and late postnatal parental depression was calculated within a 95% CI, and a threshold of 2-sided *P* ≤ .05 was established as statistical significance using the metaprop test in Stata. The metaprop command incorporates a Freeman-Tukey double arsine transformation of the prevalence data. A random-effects meta-analysis model was chosen to account for diverse study designs, as it assumes that the prevalence estimate will vary across these studies. Statistical heterogeneity was estimated using the *I*^2^ statistic, which determines how much variation is because of true study differences and not because of chance. We performed a sensitivity analysis to assess whether the meta-analysis results were influenced by the exclusion of 2 outlying studies.

## Results

### Search Results

**Figure 1.**
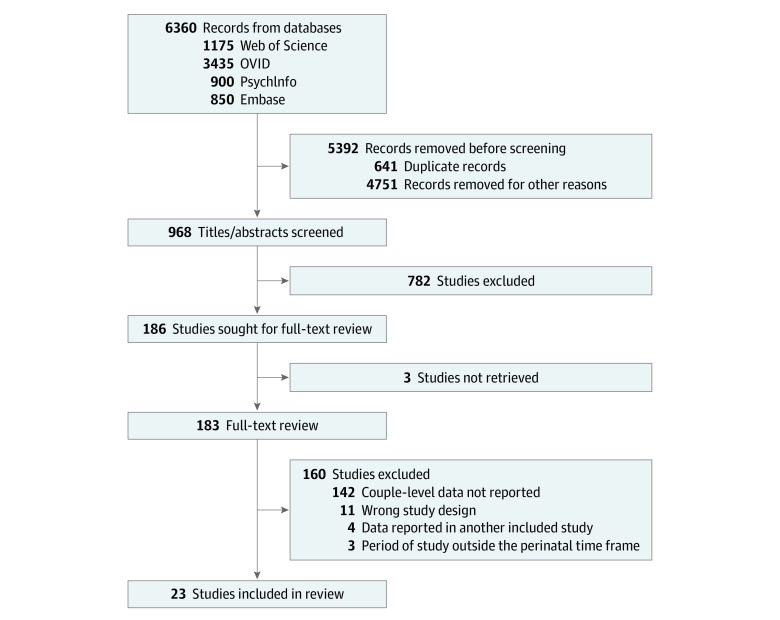
Preferred Reporting Items for Systematic Reviews and Meta-anlyses Flow Diagram of Search Results and Review of Studies for Inclusion

The systematic search yielded 6360 references of which 23 studies (29 286 couples) met criteria for inclusion in the review. Figure 1 displays the study flowchart of the search results. The primary reviewer (K.L.S.) screened all studies and a second reviewer screened 50% with 99% consensus between reviewers. Full-text review was performed for 183 studies (Figure 1).

### Description of Studies

Most studies were either cross-sectional or cohort studies. Six studies used data from larger cohorts at 1 or more points in time, providing cross-sectional analyses.^17,18,19,20,21,22^ Sample size varied from 51 couples to more than 12 000 couples.^16,17,18,19,20,21,22,23,24,25^

Most studies assessed postnatal depression. In 6 studies (26%), data were presented for the antenatal period.^23,24,25,26,27,28^ Two studies reported couple-level data for both antenatal and postnatal time points.^25,27^ All studies were performed in high-income countries (according to World Bank gross national income per capita criteria),^29^ apart from 1 that was performed in Brazil.^30^

All studies used validated screening tools to assess risk of mood disorders. The most common tool was the EPDS, used for both mothers and fathers in 23 studies^17,18,19,20,21,22,23,24,25,26,27,28,30,31,32,33,35,36,37,38,39,40,41^ (74%). Although the EPDS has been validated in many different settings, the cutoff varies, and this is reflected in the range of cutoffs used (8-13). The Center for Epidemiologic Studies Depression Scale was used in 5 studies,^22,27,28,31,32^ while the Beck Depression Inventory, Second Edition and General Health Questionnaire were used by 1 study.^33^ One study used the Kessler-6 scale, which measures nonspecific distress or mental illness.^34^ Studies^17,18,19,20,21,22,23,24,25,26,27,28,30,31,32,33,35,36,37,38,39,40,41^ were grouped according to the outcome studied and key details are listed in the Table.

**Table.** Studies Included in the Systematic Review

Variable and source (country)	Study aim(s)	Study design	Time of assessment	Couples, No.	Screening tool	Cutoff	Prevalence of depression in both parents, %
Antenatal depression							
Raskin et al,^27^ 1990 (US)	Describe the pattern of antenatal and postnatal depression in participants from childbirth classes	Cohort	Third trimester	86	CES-D	≥16	4.7
Escriba-Agueiret al,^26^ 2008 (Spain)	Determine whether there are gender differences in effect of psychosocial and personal factors on depression; recruited women attending clinic during their third trimester	Cross-sectional	Third trimester	664	EPDS-Spanish	≥13 (women); ≥11 (men)	1.5
Conde et al,^25^ 2011 (Portugal)	Examine the interaction between attachment style and partner support with perinatal mood disorders among parents recruited from antenatal clinic	Cohort	Second trimester	63	EPDS-Portuguese	≥10	3.2
Della Vedova et al,^24^ 2019 (Italy)	Examine the relationship between prenatal attachment and mood disorders among couples recruited from childbirth classes	Cross-sectional	Third trimester	93	EPDS-Italian	≥10	0
Kiepura et al,^23^ 2020 (Poland)	Estimate the prevalence of antenatal depression and anxiety among first-time parents recruited from antenatal classes	Cross-sectional	Third trimester	169	EPDS	≥12	1.2
Mangialavori et al,^28^ 2021 (Italy)	Examine the association between perceived stress, dyadic satisfaction, and antenatal depression among a community sample of first-time parents	Cross-sectional	Third trimester	138	CES-D	≥16	4.37
Early postnatal depression							
Raskin et al,^27^ 1990 (US)	Describe the pattern of antenatal and postnatal depression in participants from childbirth classes	Cohort	8 wk post partum	86	CES-D	≥16	4.7
Ballard et al,^37^ 1994 (UK)	Examine whether postnatal depression in couples recruited from maternity ward is higher than expected by chance	Cohort	6 wk post partum	178	EPDS	≥13	4.49
Lane et al,^36^ 1997 (Ireland)	Determine the correlates and variables associated with postnatal depression among a sample of women recruited in postpartum ward	Cross-sectional	6 wk post partum	173	EPDS	≥13	0.58
Soliday et al,^31^ 1999 (US)	Examine postnatal functioning in both parents and the outcomes associated with children’s development. Recruited from childbirth class, 10 secondary recruits	Cross-sectional	4-6 wk post partum	51	CES-D	≥17	19.6
Pinheiro et al,^30^ 2006 (Brazil)	Estimate the prevalence of paternal postnatal depression, and the association with maternal depression in a random sample of new parents	Cross-sectional	6-12 wk post partum	386	BDI-Portuguese	≥19	3.37
Matthey et al,^33^ 2000 (Australia)	Estimate the prevalence of postnatal depression in first-time parents recruited from clinic in second trimester	Cohort	6 wk post partum	157	EPDS, BDI, GHQ	EPDS ≥13; BDI ≥10 (women) and ≥7 (men); GHQ ≥8	2.77
Currò et al,^39^ 2009 (Italy)	Determine the role of the pediatrician in detecting postnatal depression. Participants recruited by pediatricians at first well-baby visit	Cross-sectional	2-8 wk post partum	497	EPDS	≥10 (women); ≥8 (men)	6.2
Nishimura et al,^32^ 2010 (Japan)	Determine risk factors for depression and EPDS cutoff score among fathers; mothers recruited at the postnatal check	Cross-sectional	4-6 wk post partum	129	CES-D, EPDS	CES-D ≥16; EPDS ≥9 (women); ≥8 (men)	6.2
Conde et al,^25^ 2011 (Portugal)	Examine the interaction between attachment style and partner support with perinatal mood disorders among parents recruited from antenatal clinic	Cohort	12 wk post partum	63	EPDS-Portuguese	≥10	1.6
Kerstis et al,^21^ 2012 (Sweden)	To determine whether parental relationship discord is associated with postpartum depression among a cohort of new parents attending child health centers	Cross-sectional analysis of cohort study	12 wk post partum	249	EPDS-Swedish	≥10	2.4
Anding et al,^35^ 2015 (Germany)	Determine prevalence of depressive symptoms in both parents using data from the German Midwife Prevention Study	Cross-sectional	2 wk post partum	276	EPDS-German	≥13 (women); ≥11 (men)	2.9
Kerstis et al,^20^ 2016 (Sweden)	Describe the association between parental depressive symptoms and bonding with infant among a population-based cohort recruited from hospital	Cross-sectional analysis of cohort study	6 wk post partum	727	EPDS-Swedish	≥10	1.38
Massoudi et al,^40^ 2016 (Sweden)	Estimate the prevalence and correlation of maternal and paternal postnatal depression among a population-based sample of parents	Cross-sectional	12 wk post partum	858	EPDS-Swedish	≥12	1.5
Leung et al,^19^ 2017 (Canada)	Determine the variables associated with postnatal depression among couples using data from the APrON study	Cross-sectional analysis of cohort study	12 wk post partum	846	EPDS	≥10 (women); ≥9 (men)	2.3
Clavenna et al,^41^ 2017 (Italy)	Determine the prevalence of parental depression among parents attending pediatric visits	Cross-sectional	8-12 wk post partum	1410	EPDS-Italian	≥13	0.6
Nishigori et al,^17^ 2019 (Japan)	Determine the prevalence of parental depression using data from the Japan Environment and Children’s Study	Cross-sectional	4 wk post partum	1023	EPDS-Japanese	≥9 (women), ≥8 (men)	2.24
Nakamura et al,^18^ 2020 (France)	Examine the association between support during pregnancy and parental postnatal depression using data from the ELFE study	Cross-sectional analysis of cohort study	8 wk post partum	12 386	EPDS	≥12 (women), ≥10 (men)	1.35
Takehara et al,^38^ 2020 (Japan)	Examine the prevalence of psychological distress in the first postnatal year using data from Comprehensive Survey of Living Conditions	Cross-sectional	0-12 wk post partum	734	Kessler-6	≥9	3.4
Late postnatal depression							
Ballard et al,^37^ 1994 (UK)	Examine whether postnatal depression in couples recruited from maternity ward is higher than expected by chance	Cohort	6 mo post partum	148	CES-D	≥16	4.7
Paulson et al,^22^ 2006 (US)	Determine whether postnatal depression is associated with parenting behaviors using data from early childhood longitudinal study	Cross-sectional analysis of cohort study	9 mo post partum	5089	CES-D	≥10	2.9
Matthey et al,^33^ 2000 (Australia)	Estimate the prevalence of postnatal depression in first-time parents recruited from clinic in second trimester	Cohort	12 mo post partum	146	EPDS, BDI, GHQ	EPDS ≥13; BDI ≥10 (women) and ≥8 (men); GHQ ≥8	6.57
Nishigori et al,^17^ 2019 (Japan)	Determine the prevalence of parental depression using data from the Japan Environment and Children’s Study	Cross-sectional analysis of cohort study	6 mo post partum	1330	EPDS-Japanese	≥9 (women); ≥8 (men)	2.33
Takehara et al,^38^ 2020 (Japan)	Examine the prevalence of psychological distress in the first postnatal year using data from Comprehensive Survey of Living Conditions	Cross-sectional	>3-6 mo	872	Kessler-6	≥9	1.8
			>6-9 mo	937			4.5
			>9-12 mo post partum	971			3.6
Perinatal anxiety							
Kiepura et al,^23^ 2000 (Poland)	Estimate the prevalence of antenatal depression and anxiety among first-time parents recruited from antenatal classes	Cross-sectional	Third trimester	169	STAI	Trait ≥8; state ≥7 (STEN)	NR
Conde et al,^25^ 2011 (Portugal)	Examine the interaction between attachment style and partner support with perinatal mood disorders among parents recruited from antenatal clinic	Cohort	Second trimester	63	STAI	≥45	4.8
			3 mo post partum				3.2
Della Vedova et al,^24^ 2019 (Spain)	Examine the relationship between prenatal attachment and mood disorders among couples recruited from childbirth classes	Cross-sectional	Third trimester	93	EPDS-3A	≥6	0

Abbreviations: APrON, Alberta Pregnancy Outcomes and Nutrition; BDI, Beck Depression Inventory; CES-D, Center for Epidemiologic Studies Depression Scale; ELFE, Etude Longitudinale Française depuis l'Enfance (French longitudinal study of children); EPDS, Edinburgh Postnatal Depression Scale; GHQ, General Health Questionnaire; NR, not reported; STAI, State-Trait Anxiety Inventory; STEN, standard tens score.

### Quality Assessment

Most studies were judged to be of moderate quality. Participants were commonly recruited from antenatal or postnatal clinics or childbirth classes, which is appropriate as expectant or new parents are the population of interest. Most studies provided detailed inclusion and exclusion criteria, limiting participants to low-risk parents. Sample sizes were fairly small (<196 couples) in 10 studies (43%), and 1 study used secondary recruitment to increase the number of participants, potentially leading to selection bias.^31^ Response rates were greater than 60% in 19 of the 23 studies. Two studies had suboptimal response rates, and this was addressed in their discussion as a source of nonresponse bias.^32,33^ Two studies did not provide adequate information to determine response rates.^17,35^ See eAppendix 2 in the Supplement for full quality assessment.

### Outcome Measures

#### Antenatal Depression

**Figure 2.**
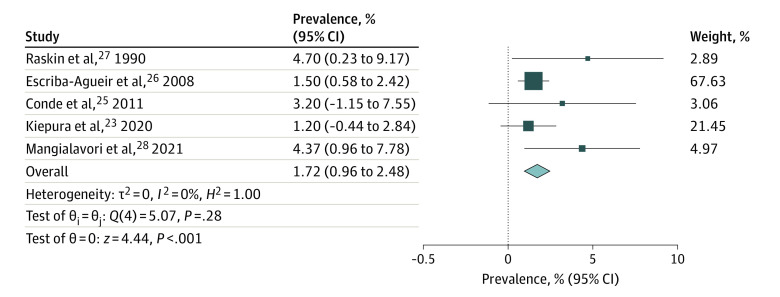
Prevalence of Antenatal Depression in Parental Dyads Forest plot shows the pooled prevalence of antenatal depression in parental dyads according to random-effects meta-analysis. Size of boxes is a visual representation for the weight of that study in the meta-analysis. Whiskers indicate the 95% confidence interval for the prevalence in each study. The diamond indicates the pooled prevalence (%) according to the random-effects meta-analysis estimate of effect size. Diameter of the diamond reflects the 95% CI for the estimate.

Prevalence of antenatal depression in both members of a parental dyad ranged from 0% to 4.7%, with study sample sizes between 63 and 664 couples (Table). The study by Della Vedova et al^24^ was an outlier, as no men had antenatal depression according to screening with the abbreviated EPDS 3-A. Therefore, data from the 5 remaining studies (1120 couples) assessing antenatal parental depression were meta-analyzed, yielding a pooled prevalence of 1.72% (95% CI, 0.96%-2.48%; *P* < .001; *I*^2^ = 0%) (Figure 2).

#### Postnatal Depression

Eighteen studies examined depression in the early postnatal period (≤12 weeks after delivery). Five studies assessed parental depression in the later postnatal period (>3-12 months).

##### Early Postnatal Depression

**Figure 3.**
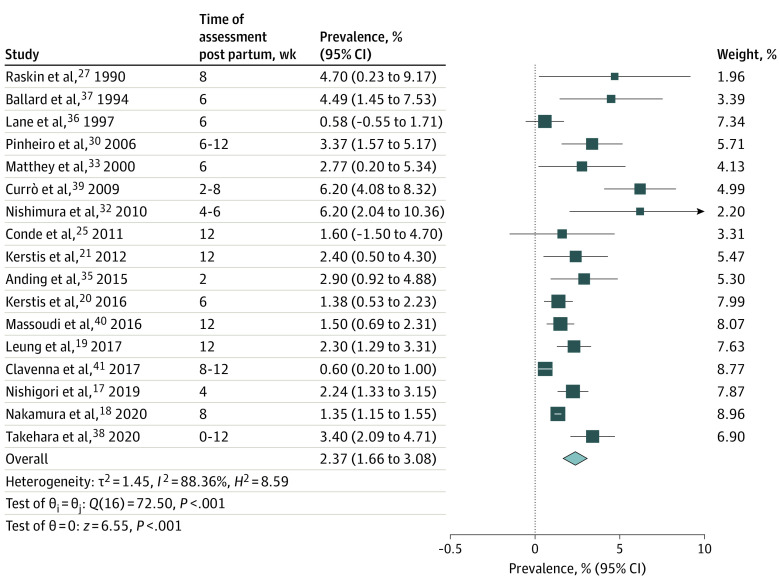
Prevalence of Early Postnatal Depression in Parental Dyads Forest plot shows the pooled prevalence of early postnatal depression (0-12 weeks after childbirth) in parental dyads according to random-effects meta-analysis. Size of boxes is a visual representation for the weight of that study in the meta-analysis. Whiskers indicate the 95% confidence interval for the prevalence in each study. The diamond indicates the pooled prevalence (%) according to the random-effects meta-analysis estimate of effect size. Diameter of the diamond reflects the 95% CI for the estimate.

Studies assessing depression up to 12 weeks post partum provided data from 20 229 couples. Point prevalence for early postnatal depression in both parents varied from 0.58% to 19.6%. Lane et al^36^ assessed postnatal depression both at 3 days and 6 weeks post partum. Mild mood changes in the first 2 weeks post partum can be common in women and are usually due to hormonal changes; therefore, measurements at 6 weeks post partum were used for the analysis. Soliday et al^31^ used the CES-D to assess depression in 51 couples 4 to 6 weeks after childbirth. Almost 20% of couples met criteria for postnatal depression. This prevalence was far higher than values reported by other studies and was subject to selection bias as the authors used secondary recruitment for 10 couples. This study was, therefore, excluded from the meta-analysis. Random-effects meta-analysis of the remaining 17 studies (20 178 couples) yielded a pooled prevalence of 2.37% (95% CI, 1.66%-3.08%; *P* < .001; *I*^2^ = 88.36%) for early postnatal depression in parental dyads (Figure 3).

##### Late Postnatal Depression

**Figure 4.**
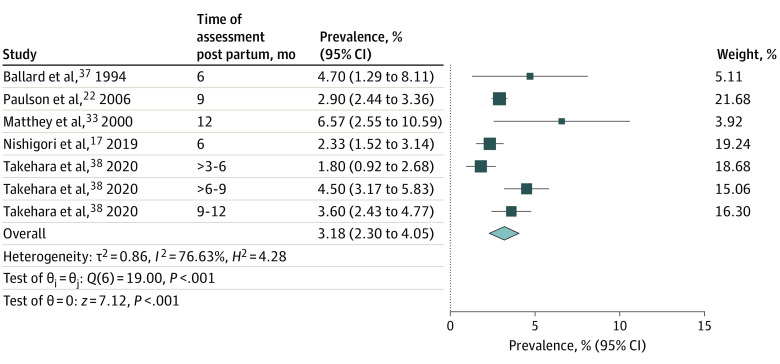
Prevalence of Late Postnatal Depression in Parental Dyads Forest plot shows the pooled prevalence of late postnatal depression (3-12 months after childbirth) in parental dyads according to random-effects meta-analysis. Size of boxes is a visual representation for the weight of that study in the meta-analysis. Whiskers indicate the 95% confidence interval for the prevalence in each study. The diamond indicates the pooled prevalence (%) according to the random-effects meta-analysis estimate of effect size. Diameter of the diamond reflects the 95% CI for the estimate.

Five studies assessed parental depression in the late postnatal period (>3-12 months). This represents data from 9493 couples, and point prevalence values ranged from 1.8% to 4.7%.^17,22,33,37,38^ Random-effects meta-analysis yielded a pooled prevalence of 3.18% (95% CI, 2.3-4.05; *P* < .001; *I*^2^ = 76.63) for late postnatal depression in parental dyads (Figure 4).

#### Anxiety

Three studies addressed parental perinatal anxiety, recruiting a low-risk group of mostly first-time parents from antenatal clinics or childbirth classes. Conde et al^25^ evaluated anxiety among Portuguese parents using the State-Trait Anxiety Inventory self-report scale, and rates of perinatal anxiety were high for both mothers (19%) and fathers (7.9%-11%). Kiepura et al^23^ also used the State-Trait Anxiety Inventory, and almost 10% of men and 7.7% of women met criteria for elevated state anxiety. However, in the study by Della Vedova et al,^24^ no men met criteria for perinatal anxiety. In contrast, a recent meta-analysis estimated the prevalence of perinatal paternal anxiety to be 10.69% (95% CI, 8.14%-13.91%).^42^ Further research is needed to determine the prevalence of parental perinatal anxiety, and to identify the ideal screening tool for use in this population.

#### Related Factors

The association between mood disorders in both parents and other covariates was not uniformly assessed in included studies, precluding statistical analysis. A narrative summary is presented here. In 3 studies,^17,19,30^ maternal antenatal depression was associated with increased risk of postnatal depression in both members of the parental dyad. Socioeconomic factors, such as unemployment, longer paternal working hours, and low income, were also associated with depression in couples.^35,37,38^ Relationship issues, such as low marital satisfaction, frequent quarrels, or perceived low support, were associated with mood symptoms in both parents.^21,26^

### Sensitivity Analyses

Two studies were excluded from the primary analysis as they were significant outliers (Della Vedova et al^24^ and Soliday et al^31^). A sensitivity analysis including these 2 studies did not significantly change the results of the primary meta-analysis (see eFigure 1 and eFigure 2 in the Supplement for primary and secondary analyses for antenatal depression, respectively; see eFigure 3 and eFigure 4 in the Supplement for primary and secondary analyses for early postnatal depression, respectively ). Subgroup analyses were performed to determine potential contributors to heterogeneity. The depression screening tool varied among studies, with a majority using the EPDS. Subgroup analysis demonstrated a difference in the prevalence of postnatal depression between studies that used the EPDS and those that did not (2.22% [95% CI, 1.5-2.95] vs 3.26% [95% CI, 2.56-3.96]; *P* = .04) (eFigure 5 in the Supplement).

## Discussion

The pooled prevalence of antenatal depression in parental dyads (mothers and fathers) was 1.72%. This estimate is limited by the small number of studies. Previous studies demonstrate that parents experiencing depression during pregnancy are more likely to experience postnatal depression. According to longitudinal cohort data, the odds of postnatal depression (measured by EPDS score) have been shown to increase by more than 3-fold for women with antenatal depression.^19^ This association was even more striking among men, with an odds ratio of 9.11 (95% CI, 5.40-15.4; *P* < .001).^19^ These findings underscore the need to address antenatal depression in new and expectant parents. However, the limited data on antenatal mood disorders within the parental dyad suggests that future research in this area is needed.

The prevalence of early postnatal depression (0-12 weeks after delivery) in both parents was estimated to be 2.37%. In high-income countries such as the US and the UK, more than 80% of women and more than 70% of men become parents.^43,44,45,46^ Therefore, with prevalence rates of 2% to 3% for depression in both members of the parental dyad, the potential burden of disease is considerable.

Perinatal depression can follow a protracted course; most men and women who have depressive symptoms at 4 and 8 weeks post partum continue to have symptoms at 6 months post partum, and some develop symptoms in the later postnatal period.^9^ In this review, the pooled prevalence of parental depression was higher in the later postnatal period (>3-12 months) (3.18% vs 2.37%), although the 95% CIs overlapped. Future research should determine the longitudinal course of perinatal mood disorders coexisting in both parents, which may change clinical practice. The clinical focus on postnatal depression usually centers on the first 12 weeks post partum, reflected in practice guidelines.^47^ However, our findings suggest that clinical attention to perinatal mood disorders may need to extend beyond the early postnatal period.

### Related Factors

This review assessed parental perinatal mood disorders in low-risk pregnancies. Complications in pregnancy such as stillbirth, preterm labor, or HIV are likely factors associated with risk for perinatal mood disorders. However, the impact of pregnancy complications on the prevalence of mood disorders in both parents was beyond the scope of this review.

Factors such as low relationship satisfaction and socioeconomic hardship were associated with higher prevalence of mood disorders in parental dyads.^21,26,35,37,38^ This highlights the need to consider health and well-being in a wider context. Focusing on the social determinants of health as well as the relationship between expectant parents may identify couples at risk for perinatal mood disorders. Future research should examine factors associated with increased risk for mood disorders in a parental dyad, and whether they differ from factors associated with risk for isolated maternal or paternal mood disorders.

### Potential Impacts

According to the results of this review, both members of the parental dyad experience perinatal mood disorders in up to 3 out of 100 couples. This has consequences for the individual’s health and well-being, their ability to parent their child, as well as the mental and physical health of their children.^1,10^ Parental perinatal mood disorders also increase relationship discord and risk of separation.^48^

There is evidence that a healthy father-child relationship can mitigate against poorer child outcomes in cases of maternal depression.^49,50^ In cases of paternal depression, a healthy mother-child relationship will likely offer similar protection.^51^ However, when both parents are depressed, this buffering effect is lost, further increasing the risk for poor mental and physical health outcomes for their child. Awareness of the coexistence of common mental health disorders in both members of a parental dyad allows for a paradigm shift in the provision of perinatal care. In the UK, there is usually a single postnatal visit for mothers during the first 6 to 8 weeks after childbirth.^47^ However, review of electronic health records suggests that up to 40% of new mothers may not have this consultation,^52^ and fathers are seldom included. Perhaps it is time to rethink the current model of postnatal care and move toward a holistic model of care that better supports both parents during the antenatal period and extends beyond the early perinatal period.

### Limitations

This study had limitations. Although we sought to investigate both anxiety and depression, there were limited data available on perinatal anxiety in both mothers and fathers. Anxiety and depression often coexist in the general population, with up to 67% of patients with major depression meeting criteria for an anxiety disorder.^53^ A recent meta-analysis reported the prevalence of maternal comorbid anxiety and depression to be 9.5% during pregnancy and 8.2% in the first 6 months post partum.^54^ Paternal perinatal anxiety rates are higher than in the general population (10.69% vs 3.8%).^42^ This illustrates the need for future research on anxiety in parental dyads during the transition to parenthood.

There were data from 15 different countries, improving the generalizability, particularly to other high-income countries. However, the inclusion of studies from several different countries using a range of screening tools contributes to the observed heterogeneity. A random-effects meta-analysis was adopted to account for the between-study heterogeneity^55^; however, this model gives a higher weight to smaller studies, possibly limiting the validity of the results obtained. Smaller sample size also means that those study estimates are less precise.

Most of the studies used cross-sectional data, limiting the analysis of covariates that may be associated with the prevalence of perinatal parental mood disorders. Although several studies excluded patients with a history of mental health problems, this was not done uniformly. Thus, it is impossible to identify true incident cases. Although some studies followed a cohort of patients, there were insufficient data to comment on the course of perinatal mood disorders for the parental dyad. To understand the trajectory of perinatal depression in the parental dyad, longitudinal data following large cohorts will be required.

Most studies were conducted in high-income countries; therefore, the findings may not apply to low- or middle-income countries. Given that rates of maternal depression and anxiety are higher in low- or middle-income countries than high-income countries,^1,56^ further research should address the prevalence of mood disorders in parental dyads in this context. Additionally, the systematic search did not yield any studies that examined perinatal mood disorders in both parents outside of a heteronormative context. Future research should consider the full spectrum of family-building.

## Conclusions

This review found that both mothers and fathers experienced depression in up to 3% of couples. Given the presence of substantial heterogeneity, conclusions are tentative. Further research should examine the coexistence of mood disorders in new or expectant parents, and the ideal screening tool, particularly for new or expectant fathers.

## Notes

Supplement. **eAppendix 1.** Inclusion and Exclusion Criteria **eAppendix 2. **Quality Assessment Using Joanna Briggs Institute Criteria for Prevalence Studies **eFigure 1. **Forest Plot of Prevalence of Parental Antenatal Depression (Primary Analysis) **eFigure 2. **Forest Plot of Prevalence of Parental Antenatal Depression (Secondary Analysis) **eFigure 3. **Forest Plot of Prevalence of Early Postnatal Depression (Primary Analysis) **eFigure 4. **Forest Plot of Prevalence of Early Parental Postnatal Depression (Secondary Analysis) **eFigure 5. **Subgroup Analyses Based on Time of Postpartum Assessment and Depression Screening Tool Used
