# Supplementary material for: Prevalence of Perinatal Depression and Anxiety in Both Parents: A Systematic Review and Meta-analysis
Source: JAMA Netw Open. 2022 Jun 24;5(6):e2218969. doi: 10.1001/jamanetworkopen.2022.18969 (PMC9233234; doi:10.1001/jamanetworkopen.2022.18969)
Supplement: Supplement. — eAppendix 1. Inclusion and Exclusion Criteria eAppendix 2. Quality Assessment Using Joanna Briggs Institute Criteria for Prevalence Studies eFigure 1. Forest Plot of Prevalence of Parental Antenatal Depression (Primary Analysis) eFigure 2. Forest Plot of Prevalence of Parental Antenatal Depression (Secondary Analysis) eFigure 3. Forest Plot of Prevalence of Early Postnatal Depression (Primary Analysis) eFigure 4. Forest Plot of Prevalence of Early Parental Postnatal Depression (Secondary Analysis) eFigure 5. Subgroup Analyses Based on Time of Postpartum Assessment and Depression Screening Tool Used [file jamanetwopen-e2218969-s001.pdf]

Supplemental Online Content

Smythe KL, Petersen I, Schartau P. Prevalence of perinatal depression and anxiety in both parents: a systematic review and meta-analysis. *JAMA Netw Open*. 2022;5(6):e2218969. doi:10.1001/jamanetworkopen.2022.18969

eAppendix 1. Inclusion and Exclusion Criteria
eAppendix 2. Quality Assessment Using Joanna Briggs Institute Criteria for Prevalence Studies
eFigure 1. Forest Plot of Prevalence of Parental Antenatal Depression (Primary Analysis)
eFigure 2. Forest Plot of Prevalence of Parental Antenatal Depression (Secondary Analysis)
eFigure 3. Forest Plot of Prevalence of Early Postnatal Depression (Primary Analysis)
eFigure 4. Forest Plot of Prevalence of Early Parental Postnatal Depression (Secondary Analysis)
eFigure 5. Subgroup Analyses Based on Time of Postpartum Assessment and Depression Screening Tool Used

This supplemental material has been provided by the authors to give readers additional information about their work.

© 2022 Smythe KL et al. *JAMA Network Open.*

eAppendix 1: Inclusion and Exclusion Criteria

INCLUSION	EXCLUSION
Assesses mothers and fathers together	High-risk pregnancy or complicated birth
Adults ≥18 years	Anxiety related to prenatal diagnosis
Depression and/or anxiety diagnosed based on validated screen (risk)	Assessment only within first 72 hours of birth
OR clinical criteria (ICD-10 code; DSM-V criteria)	Trials
OR prescription of antidepressant/anxiolytic	Studies done for validation of screening tool
Diagnosis during pregnancy and/or up to 1 year postpartum	Review articles
Observational studies published from 1990-2021	Full text unavailable
English language	
Any country	

© 2022 Smythe KL et al. *JAMA Network Open.*

eAppendix 2: Quality Assessment Using Joanna Briggs Institute Criteria for Prevalence Studies

Title	Lead author, year	Was the sample frame appropriate to address the target population?	Were study participants sampled in an appropriate way?	Was the sample size adequate?	Were the study subjects and the setting described in detail?	Was the data analysis conducted with sufficient coverage of the identified sample?	Were valid methods used for the identification of the condition	Was the condition measured in a standard, reliable way for all participants?	Was there appropriate statistical analysis	Was the response rate adequate, and if not, was the low response rate managed appropriately?
Patterns of depressive symptoms in expectant and new parents	Raskin et al 1990	Yes	Unclear	No	Limited	Yes	Yes	Yes	Yes	Yes
Prevalence of Postnatal Psychiatric Morbidity in Mothers and Fathers	Ballard et al 1994	Yes	Yes	No	Yes	Yes	Yes	Yes	Yes	Yes
Postnatal depression and elation among mothers and their partners: Prevalence and predictors	Lane et al 1997	Yes	Yes	No	Yes	Yes	Yes	Yes	Yes	Yes
Postpartum affect and depressive symptoms in mothers and fathers	Soliday et al 1999	Yes	Secondary recruitment of 10 couples; limited detail on inclusion/ excusion	No	Yes	Yes	Yes	Yes	Yes	Yes
Paternal and maternal depressed mood during the transition to parenthood	Matthey 2000	Yes	Yes	No	Yes	Yes	Yes	Yes	Yes	Borderline (59%); yes (compared nonrespondents to those who responded)
Is paternal postpartum depression associated with maternal postpartum depression? Population-based study in Brazil	Pinheiro et al 2006	Yes	Yes	Yes	Yes	Yes	Yes	Yes	Yes	Yes
Individual and combined effects of postpartum depression in mothers and fathers on parenting behavior	Paulson et al 2006	Yes	Yes	Yes	Yes	Yes	Yes	Yes	Yes	Yes

© 2022 Smythe KL et al. *JAMA Network Open.*

Factors related to depression during pregnancy: are there gender differences?	Escriba-Agueir et al 2008	Yes	Yes	Yes	Yes	Yes	Yes	Yes	Yes	Yes
The use of Edinburgh Postnatal Depression Scale to identify postnatal depression symptoms at well child visit	Currò et al., 2009	Yes	Yes	Yes	Yes	Yes	Yes	Yes	Yes	Yes
Risk factors of paternal depression in the early postnatal period in Japan	Nishimura et al 2010	Yes	Yes	No	Yes	Yes	Yes	Yes	Yes	No; discussed in limitations
Attachment style and psychological adjustment in couples	Conde et al 2011	Yes	Yes	No	Yes	Yes	Yes	Yes	Yes	Yes
The association between perceived relationship discord at childbirth and parental postpartum depressive symptoms: a comparison of mothers and fathers in Sweden	Kerstis et al 2012	Yes	Yes	Yes	Yes	Yes	Yes	Yes	Yes	Yes
Couple comorbidity and correlates of postnatal depressive symptoms in mothers and fathers in the first two weeks following delivery	Anding et al 2015	Yes	Yes	Yes	Yes	Yes	Yes	Yes	Yes	Unclear - secondary data analysis, with suboptimal response to primary data addressed by authors
Association between parental depressive symptoms and impaired bonding with the infant	Kerstis et al 2016	Yes	Yes	Yes	Yes	Yes	Yes	Yes	Yes	Yes
Fathers' depressive symptoms in the postnatal period: Prevalence and correlates in a population-based Swedish study	Massoudi et al 2016	Yes	Yes	Yes	Yes	Yes	Yes	Yes	Yes	Yes
Postnatal depression screening in a paediatric primary care setting in Italy	Clavenna et al 2017	Yes	Yes	Yes	Yes	Yes	Yes	Yes	Yes	Yes

© 2022 Smythe KL et al. *JAMA Network Open.*

**Predictors of Postpartum Depression in Partnered Mothers and Fathers from a Longitudinal Cohort**	Leung et al 2017	Yes	Yes	Yes	Yes	Yes	Yes	Yes	Yes	Yes
**Prenatal attachment, distress symptoms and psychosocial variables in a sample of Italian first-time parents**	Della Vedova et al 2019	Yes	Yes	No	Yes	Yes	Yes	Yes	Yes	Yes
**The prevalence and risk factors for postpartum depression symptoms of fathers at one and 6 months postpartum: an adjunct study of the Japan Environment & Children's Study**	Nishigori et al 2019	Yes	Yes	Yes	Yes	Yes	Yes	Yes	Yes	Unclear - rate of recruitment could not be calculated as this was adjunct study
**Antenatal depression and anxiety in primiparous Polish mothers and fathers**	Kiepura 2020	Yes	Yes	No	Yes	Yes	Yes	Yes	Yes	Yes
**Informal and formal social support during pregnancy and joint maternal and paternal postnatal depression: Data from the French representative ELFE cohort study**	Nakamura et al 2020	Yes	Yes	Yes	Yes	Yes	Yes	Yes	Yes	Yes
**Parental psychological distress in the postnatal period in Japan: a population based analysis of a national cross sectional survey**	Takehara et al 2020	Yes	Yes	Yes	Yes	Yes	Yes	Yes	Yes	Yes
**A dyadic approach to stress and prenatal depression in first-time parents: The mediating role of marital satisfaction**	Mangialavori et al 2021	Yes	Yes	No	Yes	Yes	Yes	Yes	Yes	Yes

© 2022 Smythe KL et al. *JAMA Network Open.*

Pooled Prevalence (%) of Parental Antenatal Depression		Pooled Prevalence (%) of Parental Early Postnatal Depression	
Primary Analysis	Alternative Analysis (includes data from Della Vedova et al. [2019])	Primary Analysis	Alternative Analysis (includes data from Soliday et al. [1999])
1.72% (95% CI 0.96-2.48%, p<0.001) I^2 =0%	1.64% (95% CI 0.29-3%, p=0.02) I^2 = 81.4%	2.37% (95% CI 1.66-3.08%, p<0.001) I^2=88.36%	2.47% (95% CI 1.73-3.2%, p<0.001) I^2=88.79%

eFigure 1: Forest Plot of Prevalence of Parental Antenatal Depression (Primary Analysis)

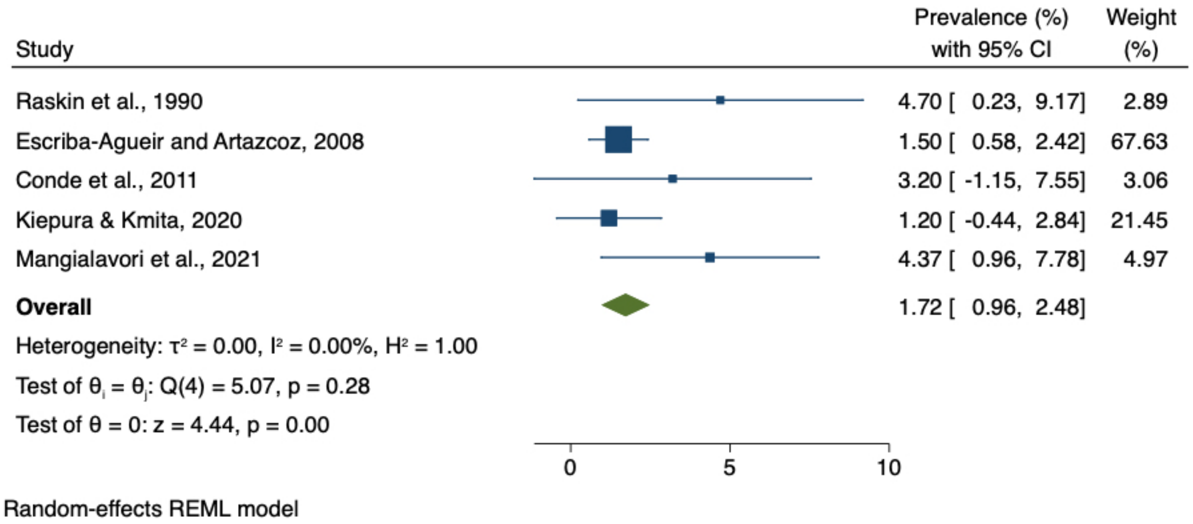

© 2022 Smythe KL et al. *JAMA Network Open.*

eFigure 2: Forest Plot of Prevalence of Parental Antenatal Depression (Secondary Analysis)

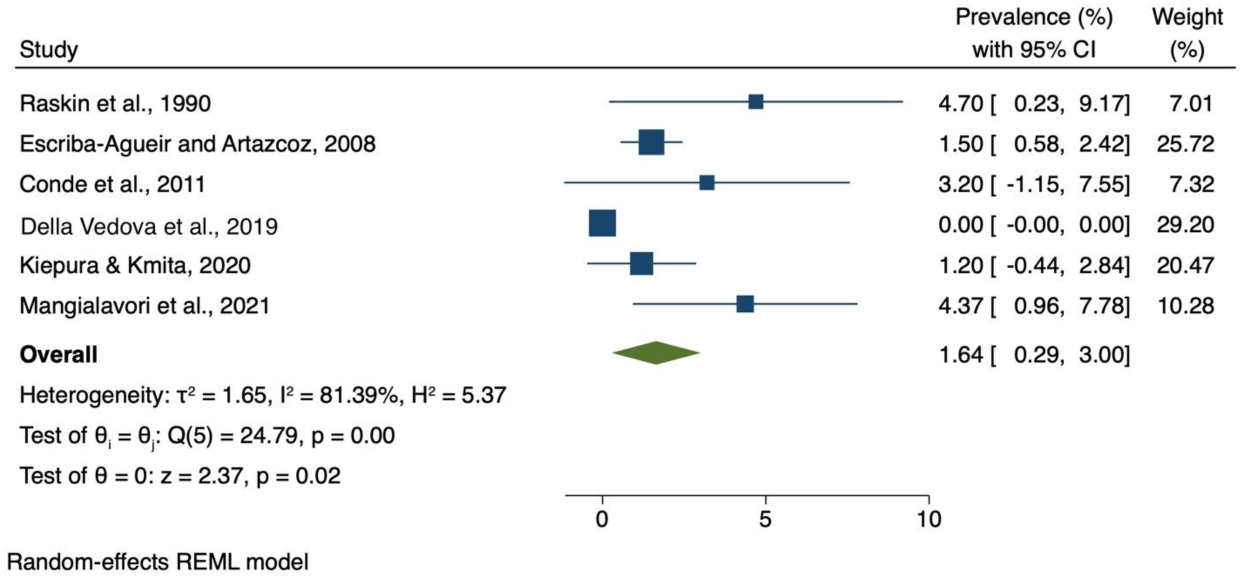

© 2022 Smythe KL et al. *JAMA Network Open.*

eFigure 3: Forest Plot of Prevalence of Early Postnatal Depression (Primary Analysis)

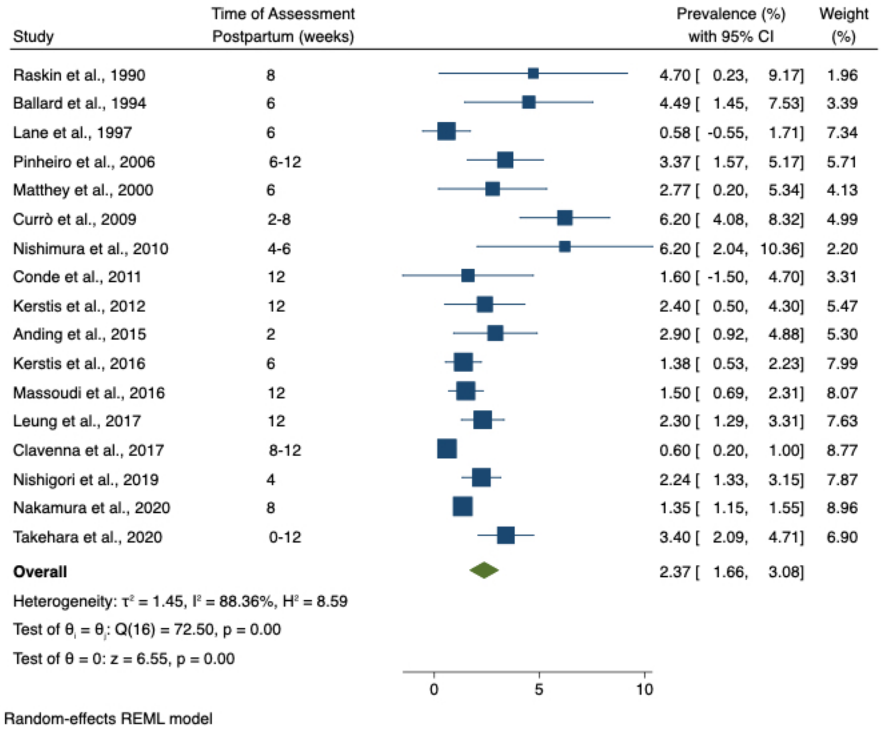

© 2022 Smythe KL et al. *JAMA Network Open.*

eFigure 4: Forest Plot of Prevalence of Early Parental Postnatal Depression (Secondary Analysis)

Study	Time of Assessment (weeks)	Prevalence (%) with 95% CI	Weight (%)
Raskin et al., 1990	8	4.70 [0.23, 9.17]	2.06
Ballard et al., 1994	6	4.49 [1.45, 7.53]	3.50
Lane et al., 1997	6	0.58 [-0.55, 1.71]	7.26
Soliday et al., 1999	4-6	19.60 [8.70, 30.50]	0.43
Pinheiro et al., 2006	6-12	3.37 [1.57, 5.17]	5.74
Matthey et al., 2000	6	2.77 [0.20, 5.34]	4.23
Currò et al., 2009	2-8	6.20 [4.08, 8.32]	5.07
Nishimura et al., 2010	4-6	6.20 [2.04, 10.36]	2.30
Conde et al., 2011	12	1.60 [-1.50, 4.70]	3.42
Kerstis et al., 2012	12	2.40 [0.50, 4.30]	5.52
Anding et al., 2015	2	2.90 [0.92, 4.88]	5.36
Kerstis et al., 2016	6	1.38 [0.53, 2.23]	7.85
Massoudi et al., 2016	12	1.50 [0.69, 2.31]	7.91
Leung et al., 2017	12	2.30 [1.29, 3.31]	7.52
Clavenna et al., 2017	8-12	0.60 [0.20, 1.00]	8.54
Nishigori et al., 2019	4	2.24 [1.33, 3.15]	7.73
Nakamura et al., 2020	8	1.35 [1.15, 1.55]	8.70
Takehara et al., 2020	0-12	3.40 [2.09, 4.71]	6.85
**Overall**		2.47 [1.73, 3.20]	

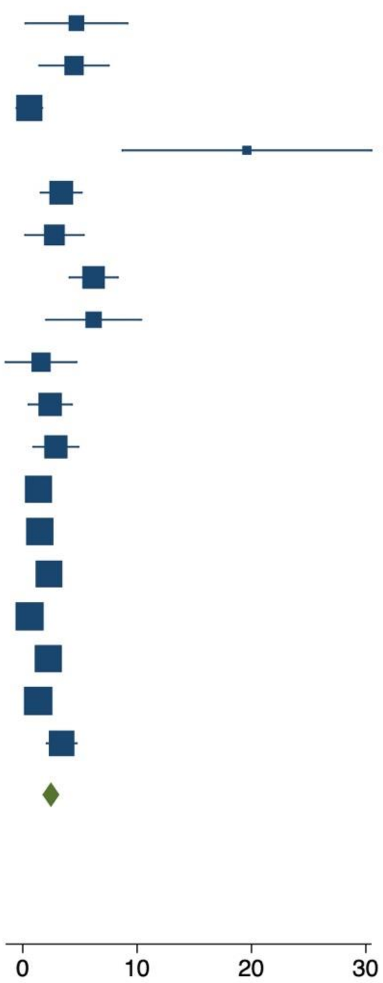

Heterogeneity: $\tau^2 = 1.60$, $I^2 = 88.79\%$, $H^2 = 8.92$

Test of $\theta_i = \theta_j$: Q(17) = 83.22, p = 0.00

Test of $\theta = 0$: z = 6.58, p = 0.00

Random-effects REML model

© 2022 Smythe KL et al. *JAMA Network Open.*

eFigure 5: Subgroup Analyses Based on Time of Postpartum Assessment and Depression Screening Tool Used

Study	K	Prevalence (%) with 95% CI	p–value
>3–12 months	7	3.18 [2.30, 4.05]	0.000
0–3 months	17	2.37 [1.66, 3.08]	0.000
Test of group differences: $Q_b(1) = 1.98$, $p = 0.16$			
Non–EPDS	9	3.26 [2.56, 3.96]	0.000
EPDS	15	2.22 [1.50, 2.95]	0.000
Test of group differences: $Q_b(1) = 4.03$, $p = 0.04$			
		2.64 [2.06, 3.23]	0.000

Heterogeneity: $\tau^2 = 1.42$, $I^2 = 88.74\%$, $H^2 = 8.88$

Test of $\theta_i = \theta_j$: $Q(23) = 150.32$, $p = 0.00$

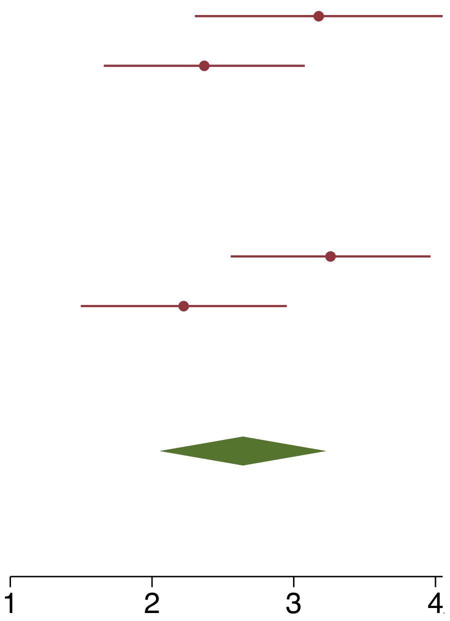

Random–effects REML model

© 2022 Smythe KL et al. *JAMA Network Open.*
